# European expert consensus on practical management of specific aspects of parathyroid disorders in adults and in pregnancy: recommendations of the ESE Educational Program of Parathyroid Disorders (PARAT 2021)

**DOI:** 10.1530/EJE-21-1044

**Published:** 2021-12-03

**Authors:** Jens Bollerslev, Lars Rejnmark, Alexandra Zahn, Ansgar Heck, Natasha M Appelman-Dijkstra, Luis Cardoso, Fadil M Hannan, Filomena Cetani, Tanja Sikjaer, Anna Maria Formenti, Sigridur Björnsdottir, Camilla Schalin-Jäntti, Zhanna Belaya, Fraser Gibb, Bruno Lapauw, Karin Amrein, Corinna Wicke, Corinna Grasemann, Michael Krebs, Eeva Ryhänen, Özer Makay, Salvatore Minisola, Sébastien Gaujoux, Jean-Philippe Bertocchio, Zaki Hassan-Smith, Agnès Linglart, Elizabeth M Winter, Martina Kollmann, Hans-Georg Zmierczak, Elena Tsourdi, Stefan Pilz, Heide Siggelkow, Neil Gittoes, Claudio Marcocci, Peter Kamenický, the 2021 PARAT Working Group, the 2021 PARAT Working Group

**Affiliations:** 1 Faculty of Medicine, University of Oslo, Oslo, Norway; 2 Section of Specialized Endocrinology, Department of Endocrinology, Medical Clinic, Oslo University Hospital, Oslo, Norway; 3 Department of Endocrinology and Internal Medicine, Aarhus University Hospital, Aarhus, Denmark; 4 Schön-Klinik Hamburg, Department of Endocrine Surgery, Hamburg, Germany; 5 Division of Endocrinology, Department of Medicine, Leiden University Medical Center (LUMC), Leiden, the Netherlands; 6 Centro Hospitalar e Universitário de Coimbra, i3S – Instituto de Investigação e Inovação em Saúde da Universidade do Porto, Porto, Portugal; 7 Nuffield Department of Women’s and Reproductive Health, University of Oxford, Oxford, UK; 8 Department of Clinical and Experimental Medicine, University of Pisa, Pisa, Italy; 9 Institute of Endocrine and Metabolic Sciences, Vita-Salute San Raffaele University, IRCCS San Raffaele Hospital, Milan, Italy; 10 Department of Endocrinology, Metabolism and Diabetes, Karolinska University Hospital, Stockholm, Sweden; 11 Endocrinology, Abdominal Center, University of Helsinki and Helsinki University Hospital, Helsinki, Finland; 12 The National Medical Research Centre for Endocrinology, Moscow, Russia; 13 Edinburgh Centre for Endocrinology & Diabetes, Royal Infirmary of Edinburgh, Edinburgh, UK; 14 Department of Endocrinology, Ghent University Hospital, Ghent, Belgium; 15 Division of Endocrinology and Diabetology, Medical University of Graz, Graz, Austria; 16 Thyroid Center, Luzerner Kantonsspital, Luzern, Switzerland; 17 Division of Rare Diseases, Department of Pediatrics, St. Josef-Hospital, Ruhr-University Bochum, Bochum, Germany; 18 Division of Endocrinology and Metabolism, Department of Internal Medicine III, Medical University of Vienna, Vienna, Austria; 19 Division of Endocrine Surgery, Department of General Surgery, Ege University Hospital, Izmir, Turkey; 20 Department of Internal Medicine and Medical Disciplines, Sapienza University of Rome, Rome, Italy; 21 Department of Digestive, Hepatobiliary and Endocrine Surgery, Paris Descartes University, Cochin Hospital, Paris, France; 22 Assistance Publique-Hôpitaux de Paris (AP-HP), Pitié-Salpêtrière Hospital, Nephrology Department, Boulevard de l’Hôpital, Paris, France; 23 Department of Endocrinology, University Hospitals Birmingham NHS Foundation Trust, Birmingham, UK; 24 Université de Paris Saclay, AP-HP, Centre de Référence des Maladies Rares du Métabolisme du Calcium et du Phosphate, Filière OSCAR, Service d’Endocrinologie et Diabète de l’Enfant, Hôpital Bicêtre Paris Saclay, Le Kremlin Bicêtre, France; 25 Department of Obstetrics and Gynecology, Medical University of Graz, Graz, Austria; 26 Reference Centre for Rare Bone, Calcium and Phosphate Disorders – University Hospital Ghent, Ghent, Belgium; 27 Center for Healthy Aging, Department of Medicine III, Technische Universität Dresden Medical Center, Dresden, Germany; 28 Endokrinologikum Göttingen, Georg-August-University Göttingen, Göttingen, Germany; 29 Université Paris-Saclay, Inserm, Physiologie et Physiopathologie Endocriniennes, Assistance Publique-Hôpitaux de Paris, Hôpital Bicêtre, Service d’Endocrinologie et des Maladies de la Reproduction, Centre de Référence des Maladies Rares du Métabolisme du Calcium et du Phosphate, Le Kremlin-Bicêtre, France

## Abstract

This European expert consensus statement provides recommendations for the diagnosis and management of primary hyperparathyroidism (PHPT), chronic hypoparathyroidism in adults (HypoPT), and parathyroid disorders in relation to pregnancy and lactation. Specified areas of interest and unmet needs identified by experts at the second ESE Educational Program of Parathyroid Disorders in 2019 were discussed during two virtual workshops in 2021 and subsequently developed by working groups with interest in the specified areas. PHPT is a common endocrine disease. However, its differential diagnosis of familial hypocalciuric hypercalcemia (FHH), the definition and clinical course of normocalcemic PHPT, and the optimal management of its recurrence after surgery represents areas of uncertainty requiring clarifications. HypoPT is an orphan disease characterized by low calcium concentrations due to insufficient PTH secretion, most often secondary to neck surgery. Prevention and prediction of surgical injury to the parathyroid glands are essential to limit the disease-related burden. Long-term treatment modalities including the place for PTH replacement therapy and the optimal biochemical monitoring and imaging surveillance for complications to treatment in chronic HypoPT need to be refined. The physiological changes in calcium metabolism occurring during pregnancy and lactation modify the clinical presentation and management of parathyroid disorders in these periods of life. Modern interdisciplinary approaches to PHPT and HypoPT in pregnant and lactating women and their newborn children are proposed. The recommendations on clinical management presented here will serve as background for further educational material aimed at a broader clinical audience and were developed with the focus on endocrinologists in training.

## Introduction

Disturbances in calcium metabolism among adults are often related to the secretion of parathyroid hormone (PTH), as in primary hyperparathyroidism (PHPT) and chronic hypoparathyroidism (HypoPT) (1). Whereas PHPT is a common endocrine disease, especially in elderly females, the latter is an orphan disorder often diagnosed in fertile life (or earlier) (2). Most of the parathyroid disorders are sporadic, with a smaller percentage being inherited or related to endocrine syndromes. The most common form of HypoPT in adults is iatrogenic, that is acquired following neck surgery (1, 2). Changes in calcium concentrations may develop abruptly (e.g. post-surgical hypoparathyroidism) or slowly, and symptoms may be non-specific or subtle leading to postponed or late diagnosis. Thus, secondary complications may exist already at the time of diagnosis, often related to non-physiologic calcium and phosphate levels (3).

Parathyroid disorders related to PTH secretion, as entities or part of syndromes (e.g. multiple endocrine neoplasia (MEN-1, MEN-2, and MEN-4)), give rise to different challenges throughout ages. For PHPT, differential diagnosis of familial hypocalciuric hypercalcemia (FHH), the definition and course of normocalcemic PHPT, and the management of PHPT in relation to syndromes or recurrence are examples of topics with unmet needs for guidance and management. HypoPT is the last endocrine-deficiency state, where hormone replacement so far has not been established as standard management (1, 4). With the recent availability of hormone replacement therapy, this orphan disease has received major attention during the past decade. In this transition period, there is a need for guidance and recommendations on long-term management of HypoPT, as well as there is a need for improvements of surgical management on the thyroid and parathyroid glands, in order to prevent HypoPT. As many patients with parathyroid disorders are diagnosed before or during fertile age, special attention should be paid to the course of pregnancy, from the planning period to the end of lactation (4, 5, 6, 7). Major calcium metabolic changes develop in pregnancy and during lactation ensuring mineralization of the skeleton in the fetus and newborns, mainly driven by the PTH-related peptide (PTHrP). PTHrP is largely produced by the placenta and mammary tissue, contributing to calcium transport over the placenta in pregnancy and into breast milk during lactation (8).

In 2018, the European Society of Endocrinology (ESE) established a specific program focusing widely on endocrine aspects of parathyroid disorders (PARAT). European experts were invited to interdisciplinary workshops to identify unmet scientific and educational needs in parathyroid diseases. The results and recommendations of the first, 2018 workshop were subsequently published in this Journal (1). During the second workshop held in June 2019, the faculty further discussed specific areas, especially within PHPT and HypoPT. The conference was based on state-of-the-art presentations by international experts followed by break-out sessions to identify the needs in each area and a further discussion in the plenum. We published the results of the workshop as a Focus Meeting Report available at the ESE homepage (https://www.ese-hormones.org/media/2141/ese-parat-focus-report-2019_v18.pdf). A main outcome of the workshop was the identification of prioritized areas of unmet needs for management and education, paving the ground for the next phase of the program.

Here, we present the consensus recommendations on the specific areas of unmet needs within parathyroid disorders identified, based on literature reviews and expert opinions developed during the third phase of the PARAT program.

## Methods

During 2021, we organized two virtual workshops, in February and September, focusing on the specified areas of interest identified at the 2019 Workshop: specific topics of PHPT and HypoPT and parathyroid disorders in relation to pregnancy. These topics were further discussed and defined at the first virtual assembly (February 2021).

Subsequently, three individual working groups reviewed literatures and based hereupon developed consensus recommendations. Literature search was performed using primary PubMed, and specific search terms were predefined according to the topic of interest. Only human studies in English language published until May 2021 (including E-Pub ahead of print) were considered. The search was primarily restricted to this millennium. However, exceptionally older original papers/case series of particular importance for specific areas were included, if the topic was not covered in more recent studies. Attention was paid to study designs focusing on randomized clinical studies (RCT) and cohort studies with or without comparison groups, whereas case reports were only included if considered of extraordinary interest, and no data from case series (cohorts) were available. To gather as much knowledge as possible, a ‘snowballing’ technique was also used, that is, literature list of identified studies was reviewed to assure the completeness of identified studies.

To finalize the consensus recommendations, findings were presented and discussed in the entire collegium of European experts at the second workshop in September 2021. This work will be the background for further educational material aimed at a broader audience as outlined below, and with a specific focus on younger endocrinologists in training.

## Primary hyperparathyroidism

PHPT is a common endocrine disease for which surgery is the only curative treatment (9). However, various aspects of PHPT are often managed based on local practices and expert opinions, rather than evidence-based recommendations (10).

### Q1 How do we differentially diagnose FHH?

**Figure 1:**
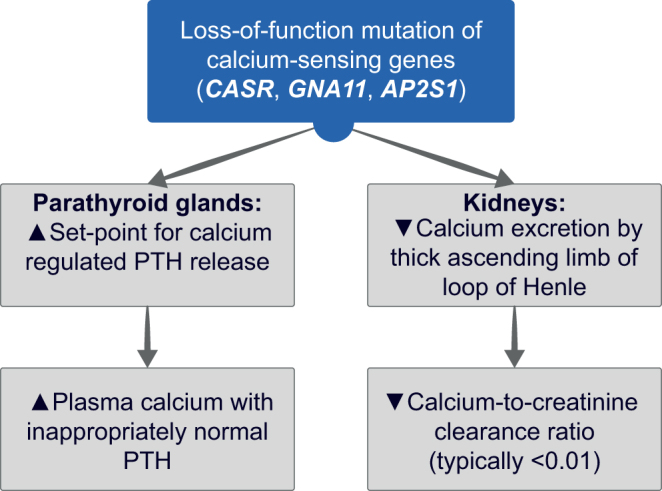
Alterations in calcium metabolism caused by familial hypocalciuric hypercalcemia (FHH). Hypercalcemia arises due to an increase in the parathyroid set-point for parathyroid hormone (PTH) release and possibly also from decreased renal calcium excretion (11, 12). Alterations in bone metabolism are not usually observed in FHH (13).

FHH is an autosomal dominant disorder affecting the calcium-sensing receptor (*CASR*) – especially at the parathyroid glands and kidneys (Fig. 1). It is characterized by lifelong non-progressive elevations of calcium concentrations, mild hypermagnesemia, normal or mildly raised PTH concentrations, and typically low urinary calcium excretion. FHH has a similar biochemical phenotype to PHPT, but FHH does not require surgical intervention. FHH is genetically heterogeneous and comprises three distinct variants, designated FHH types 1–3, which are caused by loss-of-function mutations of the *CASR*, guanine nucleotide-binding protein subunit alpha-11(*GNA11*), and adaptor protein complex-2 subunit sigma (*AP2S1*) genes, respectively (Fig. 1).

There is a role for family testing in FHH as it is a disease with a familial penetrance of >90% (i.e. proportion of hypercalcemic relatives) (14). Thus, positive family history is a key feature, and blood calcium testing of family members (especially first-degree relatives) is helpful in establishing the diagnosis.

In addition to family testing, biochemical testing contributes to distinguishing FHH from PHPT.

#### Calcium levels

The degree of hypercalcemia does not discriminate FHH from PHPT. This is due to the marked variation in calcium values in FHH, overlapping with that of PHPT (15, 16).

A review of patient records can help to establish the presence of lifelong non-progressive hypercalcemia, which is a key feature of FHH.

Although significantly higher in FHH compared to PHPT (17), serum magnesium values are usually within the reference range in FHH (16, 18), and are not useful for discriminating FHH from PHPT.

#### PTH levels

PTH levels overlap between FHH and PHPT (16, 19). However, PTH concentrations >2-fold above the upper limit of normal (ULN) are suggestive of PHPT (19).

Vitamin D insufficiency should be corrected before interpreting PTH concentrations.

#### Urine calcium excretion

This should be assessed using the calcium/creatinine clearance ratio (CCCR), which requires concurrent blood and 24 -h urine measurements.

A CCCR of <0.01 (i.e. an excretion fraction of calcium <1%) is generally considered a well-planned screening method for FHH (20). However, this cut-off is of limited clinical value due to reduced diagnostic sensitivity (only captures ~65% of FHH type 1 patients (21)) and reduced specificity (~18% of surgically confirmed PHPT cases have CCCR <0.01 (22)).

The positive predictive value of the CCCR cut-off is affected by disease prevalence. Thus, as FHH is less common than PHPT, this condition may represent a minority diagnosis in hypercalcemic patients with CCCR <0.01 (23).

CCCR has not been validated for diagnosing FHH in patients with renal impairment, vitamin D insufficiency, or pregnancy. Thiazide usage can lead to low CCCR values, as these drugs stimulate proximal tubular sodium reabsorption as a compensation of their natriuretic action in the distal tubule, resulting in enhanced proximal passive calcium transport (24). Hence, it is recommended to measure 24-h urine calcium and creatinine after withholding thiazide diuretics for at least 1 week (25).

As individual biochemical parameters cannot reliably distinguish FHH from PHPT, an algorithm termed ‘pro-FHH’ has been proposed to help discriminate between these two conditions. Pro-FHH is a risk prediction tool that incorporates calcium, PTH, a biochemical marker of bone turnover, and CCCR (26). More work is needed before pro-FHH can be advised for wider use in clinical practice.

Genetic testing is recommended for all patients with suspected FHH, as identification of a pathogenic mutation, or likely pathogenic variants, will confirm the diagnosis and differentiate between types of FHH. However, genetic testing is not a panacea. FHH type 1 is associated with a wide range of *CASR* missense variants (27), many of which are unique to individual families and are of unclear pathogenicity. Variants of unknown significance are therefore commonly detected during FHH genetic testing. Family testing and co-segregation analysis are important for determining variant pathogenicity. Genetic testing has, however, limited sensitivity, as > 25% of patients with a clinical diagnosis of FHH do not harbor germline *CASR/GNA11*/*AP2S1* genetic variation. Thus, negative genetic testing does not exclude FHH, and we recommend ongoing follow-up of mutation-negative patients, for example, historic calcium measurements, family screening, etc.

### Q2 What is normocalcemic PHPT?

**Figure 2:**
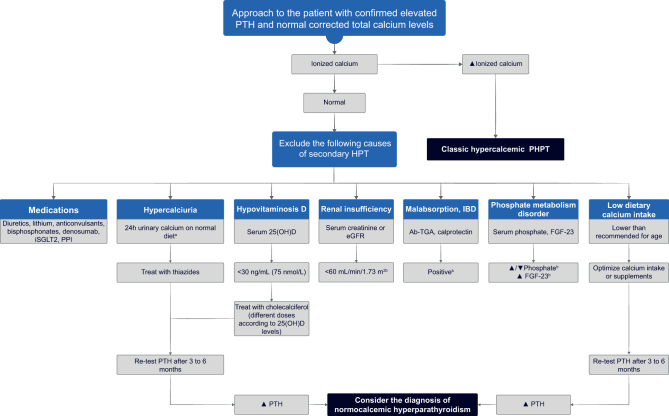
A clinical approach to patients with confirmed normocalcemic primary hyperparathyroidism. ^a^Reference range > 4 mg/kg/day, >250 mg/day in females, and >300 mg/day in males. ^b^Evaluate for these disorders and manage as appropriate. 25(OH)D, serum 25-hydroxyvitamin D; Ab-TGA, anti-tissue transglutaminase antibodies; eGFR, estimated glomerular filtration rate; FGF-23, fibroblast growth factor-23; HPT, hyperparathyroidism; IBD, inflammatory bowel disease; iSGLT2, sodium-glucose cotransporter-2 inhibitors; PHPT, primary hyperparathyroidism; PPI, proton pump inhibitor; PTH, parathyroid hormone;

Normocalcemic PHPT can be considered when there is a biochemical signature of persistently (>3 months) increased PTH levels in the setting of consistently normal total, albumin-adjusted and/or free-ionized calcium levels, based on serial laboratory measurements, when all secondary causes of PTH elevation have been excluded (28, 29). Consequently, normocalcemic PHPT represents a diagnosis of exclusion and can only be considered following a careful evaluation of causes of secondary hyperparathyroidism (Fig. 2) (30).

### Q3 What are the causes of hyperparathyroidism with normal calcium that should be excluded before considering a diagnosis of normocalcemic PHPT?

Detailed and standardized tests are required before making a diagnosis of normocalcemic PHPT.

The causes of secondary hyperparathyroidism should be excluded before diagnosing normocalcemic PHPT (Table 1).

**Table 1:** Most common causes of secondary hyperparathyroidism.

Cause of secondary hyperparathyroidism	Proposed intervention thresholds	Comments
Vitamin D deficiency	Aim for 25(OH)D concentrations of 30 ng/mL (75 nmol/L) to avoid secondary hyperparathyroidism	Re-test PTH when vitamin D replete. PTH concentrations may remain elevated for 6–12 months and optimization of calcium intake is mandatory (31)
Low-dietary calcium intake	1200 mg/day recommended for postmenopausal women, 1000 mg/day for men 51–70 years, and 1200 mg/day for older men (32)	Evaluate calcium intake using a dietary questionnaire. Patients should increase calcium intake or use calcium supplements
Hypercalciuria due to renal abnormalities	Urinary calcium excretion >250 mg/24-h (6.25 mmol/24-h) in females, >300 mg/24-h (7.5 mmol/24-h) in males, or >4 mg/kg/24-h (0.1 mmol/kg/24-h) (33, 34)	‘Thiazide challenge’ test (administer hydrochlorothiazide 25 mg twice a day for 2 weeks; check PTH levels prior to starting thiazide and after 2 weeks of therapy). PTH normalization supports renal secondary causes of PHPT (35)
Renal insufficiency	eGFR <60 mL/min/1.73 m^2^ (36, 37)	As kidney function declines, 1α-hydroxylation activity decreases and, consequently, active vitamin D levels fall, calcium levels decline, and PTH levels increase (36, 37)
Gastrointestinal disorders associated with calcium malabsorption	Celiac disease, inflammatory bowel disease, and bariatric surgery (38, 39, 40, 41)	Measure anti-tissue transglutaminase antibodies and fecal calprotectin to consider celiac disease and inflammatory bowel disease, respectively (39, 40)
Medications	Diuretics (42, 43), lithium, anticonvulsants (44, 45), bisphosphonates (46, 47), denosumab (48, 49), SGLT2 inhibitors (50, 51, 52), and proton pump inhibitors (53)	Non-thiazide diuretics can increase PTH levels (42, 43). If possible, discontinue and reevaluate PTH. Lithium therapy can raise PTH levels (44, 45). However, the decision to withdraw from therapy in these patients is difficult and should be made by a psychiatrist. Treatment with bisphosphonates or denosumab can raise PTH levels as a result of positive calcium signaling to the parathyroid glands in the context of inhibited bone resorption (7, 46, 47, 49). Bisphosphonate effects may last for a long time after discontinuation (54). Denosumab discontinuation should be avoided to prevent excessive bone loss. Recent studies showed that SGLT2 inhibitors have complex interactions with bone metabolism, including an increase in PTH (50, 51, 52)
Phosphate metabolism disorders	Hyperphosphatemia and FGF-23-mediated hypophosphatemia are both associated with secondary hyperparathyroidism (55, 56, 57)	Extracellular phosphate regulation involves changes in PTH levels. Both high and low phosphate levels may be associated with secondary hyperparathyroidism (56, 57, 58)

25(OH)D, 25-hydroxyvitamin D; eGFR, estimated glomerular filtration rate; FGF-23, fibroblast growth factor 23; PTH, parathyroid hormone; SGLT2 inhibitors, sodium-glucose cotransporter-2 inhibitors.

### Q4 What are the manifestations of normocalcemic PHPT and does it progress to hypercalcemic PHPT?

Normocalcemic PHPT may be an early biochemical manifestation of hypercalcemic PHPT (59, 60). Over an 8-year follow-up of 64 subjects identified through community screening, only 1 person developed hypercalcemic PHPT, and only 13 retained the biochemical findings of normocalcemic PHPT (61). Published series have used inconsistent definitions of normocalcemic PHPT and it is thus difficult to estimate the true prevalence and complication rate (62). Reported complication rates vary from zero in population-based studies (63, 64, 65) to 11–35% of patients with renal stones and 5–40% with low-traumatic fractures and osteoporosis, as assessed in tertiary referral centers (60, 66, 67, 68, 69). These discrepancies might reflect that many patients or cohorts are diagnosed when being evaluated for nephrolithiasis or suspected metabolic bone disease. Thus, selection bias might lead to an overestimation of the clinical impact of the disorder. It is controversial whether surgery should be considered in expert centers for selected normocalcemic PHPT patients when disease-associated progression occurs. However, evidence is limited, and the published, retrospective, surgical series do not fulfill the diagnostic criteria stated herein and above, that is repeated measurements under standardized conditions and over time (70, 71).

In summary, normocalcemic PHPT must be diagnosed by exclusion, and laboratory testing over time is necessary to distinguish normocalcemic PHPT from secondary hyperparathyroidism. Evidence on the effect of parathyroidectomy (PTX) or any other management of normocalcemic PHPT is scarce, and there are no clear data on the natural history of normocalcemic PHPT. However, unless longitudinal studies prove the opposite, established normocalcemic variant of PHPT requires standard PHPT diagnostic procedures and surgical intervention should be considered only after experienced endocrine review; in this case, only if there are compelling indications and a surgical target.

### Q5 What is the definition, prevalence, and causes of recurrent PHPT?

The most widely accepted definition of recurrent PHPT is a new finding of hypercalcemia, after a period of 6 months, in patients successfully operated and in whom normocalcemia was previously documented (72). This is distinct from persistent PHPT, defined as hypercalcemia within 6 months following surgery. The definition of recurrent PHPT primarily considers calcium levels; isolated elevation of PTH levels with normocalcemia does not represent this condition (72).

As a first step to consider when confronted with apparent recurrent PHPT, it is fundamental to confirm the diagnosis (particularly exclude FHH), repeat measurement of albumin-adjusted calcium (or free-ionized calcium) together with documentation of increased, and unsuppressed PTH concentrations (72).

There are only few data on the epidemiology of recurrent PHPT. Most authorities consider prevalence between 2.5 and 9.8% (73). Importantly, as recurrent PHPT can occur many years after initial presentation and predictive factors for recurrence are insufficiently sensitive, long-term yearly monitoring of calcium concentrations after initially successful parathyroid surgery is recommended (74).

### Q6 Do we need to act upon persistent elevations of PTH levels, despite normocalcemia?

Up to one-third of patients present elevated PTH concentrations following successful surgery for sporadic PHPT (75, 76, 77). The mechanism of this increase is unclear. In this context, elevated PTH is associated with higher preoperative PTH levels, older age, and impaired renal function. It is not, however, consistently associated with an increased risk of recurrent PHPT (75). Although elevated PTH has been associated with recurrence in some series (73, 78, 79), it is not sufficiently discriminatory to recommend routine monitoring (i.e. most individuals with elevated PTH will not have recurrence of the disease). As such, PTH should not be routinely measured in normocalcemic individuals following parathyroid surgery.

Despite recommending against routine PTH testing, clinicians will frequently be faced with the dilemma of interpreting results in this context. It is important to ensure that patients are vitamin D replete and have sufficient dietary calcium intake (Table 1), as dietary calcium deficiency can drive PTH elevation (72). Where diagnostic uncertainty persists, the suppressibility of PTH in response to a calcium load can be considered (69, 80). There are parallels between this situation and diagnosing normocalcemic PHPT, as previously discussed (Fig. 2).

In the absence of clear evidence that elevated levels of PTH with normocalcemia represent persistent or recurrent PHPT, the appropriate course is to carefully monitor serum calcium concentrations, especially in those with calcium levels toward the upper part of the reference range.

### Q7 What is the optimal work-up of patients with recurrent PHPT?

The first step in evaluating recurrent PHPT is to accurately confirm or refute the diagnosis of PHPT. This requires a thorough review of the pre-, per-, and postoperative medical, laboratory, and pathology records, exploration of the personal and family history, and repeated laboratory analyses (77, 81).

When persistent or recurrent PHPT is confirmed and a surgical approach is favored, it should be considered that about two-thirds of recurrent disease is due to a single adenoma, up to a third due to multiglandular disease, and rarely due to parathyroid carcinoma, a distinctly different profile from *de novo* PHPT (77, 81). Thus, preoperative localization procedures that are more sensitive to detect multiglandular disease and/or small lesions should be preferred (18F-fluorocholine PET/CT, with or without enhanced arterial imaging, and 4D-CT) (82, 83, 84). This is especially when conventional preoperative imaging before the first intervention was inconclusive. Moreover, localization for possible ectopic parathyroid tissue should be considered.

Importantly, an active search for potential underlying etiologies should be considered for all patients with recurrent PHPT. These could be acquired, for example, lithium-induced parathyroid hyperplasia or parathyromatosis, a rare cause of PHPT due to several hyperfunctioning parathyroid nodules in the neck and mediastinum, as a result of seeding from a ruptured parathyroid adenoma during previous parathyroid surgery (85). Other reasons relate to clinically important syndromes, such as MEN syndromes, familial isolated hyperparathyroidism, or hyperparathyroidism-jaw tumor syndrome, where genetic tests might be useful (1).

### Q8 What is the best surgical approach in patients with recurrent PHPT?

A thorough preoperative work-up is imperative, and repeat surgery should only be performed in centers with considerable experience in reoperative parathyroid surgery. Conservative medical management using cinacalcet and bone protecting agents is an adjunctive or even alternative approach to be considered, especially in patients with mild disease and/or severe comorbidities (86).

The European Society of Endocrine Surgeons suggests that challenging procedures (PHPT without unequivocal preoperative localization, hereditary variants, pediatric patients, and re-interventions) should be confined to high-volume centers performing at least 40 such procedures annually (75). Depending on the results and etiology, bilateral neck exploration or a focused minimal-invasive PTX should be performed. Surgical adjuncts, like intraoperative PTH assay and intraoperative nerve-monitoring, are recommended in cases of repeated parathyroid surgery.

### Q9 What is the risk and prevention of hypoparathyroidism following surgery for recurrent PHPT?

In the reoperative setting, the risk of transient hypoparathyroidism can be as high as 81%, while the rate of HypoPT is 3–13% (81, 87). Since there is an increased risk of hypoparathyroidism, autotransplantation of parathyroid tissue may be considered if a remaining gland is intraoperatively injured or devascularized. Recurrence of disease can occur, however, within the autotransplanted tissue. Additionally, autotransplanted grafts may fail to function in half of the cases (88), wherefore some centers have stopped performing the procedure. In accordance, centers should evaluate the procedure and the availability of cryopreservation facilities (see also ‘Chronic hypoparathyroidism in adults, Q3’).

### Q10 Why and when should calcium levels be measured after PTX?

Calcium levels should be measured postoperatively, in parallel to evaluation for hypocalcemic symptoms. For those at high risk of hungry bone syndrome (HBS) (see below), calcium levels should be checked frequently (multiple times per day) in the first postoperative days (89, 90).

To define cure of PHPT after parathyroid surgery, reestablishment of normocalcemia should last a minimum of 6 months (89, 90).

### Q11 What preoperative advice should be offered to patients awaiting PTX?

Patients with PHPT do not need to restrict dietary calcium intake. However, the normal daily intake recommendation should not be exceeded (Table 1) (32). Preoperatively, patients with vitamin D depletion are recommended to start vitamin D supplementation (86). Several studies have confirmed it to be safe (91, 92), when calcium levels are <3 mmol/L (12 mg/dL). Patients with PHPT should be advised to be well-hydrated. Patients with hypercalcemic crises require parenteral hydration and may benefit from further medical management, such as bisphosphonates, denosumab, cinacalcet, and calcitonin, or combinations of these. Prioritized surgery may be considered in this setting after medical stabilization (90).

### Q12 What causes hypocalcemia after PTX?

Postoperative hypocalcemia can be related to hypoparathyroidism or to massive transfer of calcium to bone tissue, HBS. The biochemical profile usually allows distinction between these two situations: postoperative PTH deficiency is associated with low/inappropriately ‘normal’ PTH concentrations, increased serum phosphate concentrations, and normal or elevated 24-h urinary calcium excretion with calcium replacement, whereas HBS is typically accompanied by normal or high PTH concentrations, low serum phosphate, low serum magnesium concentrations, and a low 24 -h urinary calcium excretion despite parenteral calcium replacement, as calcium is transferred into the bone tissue (93, 94). The hypocalcemia of HBS starts typically from third to fifth postoperative day (94, 95).

Hypocalcemia is usually temporary, but can be permanent if all parathyroid glands are accidentally removed or devascularized (89, 94). The risk of permanent HypoPT is increased for patients undergoing re-operation. PTH levels on the first postoperative day predict temporary hypocalcemia and HypoPT (95).

Potential risk factors for HBS are highlighted in Table 2.

**Table 2:** Potential risk factors for hungry bone syndrome.

Potential risk factors for hungry bone syndrome	Explanation
High preoperative PTH level	Sudden removal of the effect of high circulating levels of PTH on osteoclastic resorption leads to increased influx of calcium into bone (new remodeling sites) (94)
Large volume (weight and mass) of parathyroid adenoma	Positive correlation between PTH levels and volume of adenoma (93, 95)
High preoperative calcium levels	Explained as increased calcium resorption from bone and calcium reabsorption from renal tubules in case of preoperatively elevated PTH levels (95)
Radiological evidence of PHPT-related bone disease	Brown tumors, multiple fractures, osteitis fibrosa cystica as an effect of long-lasting high circulating levels of PTH on the skeleton (91, 94)
Significantly elevated alkaline phosphatase	Reflects osteoblast activity and bone formation, thus overall the state of bone turnover (91, 94)
Preoperatively low 25(OH)D concentrations	HBS develops indirectly by skeletal demineralization due to low circulating levels of 1,25(OH)_2_D with postoperative increased skeletal calcium requirements (95)

1,25(OH)_2_D, 1,25-dihydroxyvitamin D; 25(OH)D, 25-hydroxyvitamin D; HBS, hungry bone syndrome; PHPT, primary hyperparathyroidism; PTH, parathyroid hormone.

### Q13 What is optimal follow-up after (successful) PTX?

The surgical field should be checked for cervical hematoma or infection (90).

Referral for genetic panel testing is appropriate in patients <30 years. Genetic testing should be considered in PHPT patients with multiglandular disease at any age, if not performed before surgery (96).

Patients with parathyroid carcinoma or with genetic forms of PHPT (i.e. MEN types 1, 2A, 4, hyperparathyroidism-jaw tumor syndrome, and familial isolated primary hyperparathyroidism) require an individually planned follow-up in a specialized endocrine unit (1).

Although bone mineral density (BMD) improves after successful surgery (86, 97), PHPT patients with osteoporosis need an individual recommendation regarding follow-up, BMD measurement, length of calcium/vitamin D treatment, and putative need for osteoporosis medication after surgery (86, 89).

Patients with persisting hypercalcemia at 6 months after surgery should be considered for reoperation after detailed reassessment. In the presence of normocalcemia, risk of recurrence is low, but annual checks of calcium levels should be performed. If hypercalcemia emerges, PTH measurement is warranted, but as stated, routine PTH monitoring (without hypercalcemia) is not recommended (89).

## Chronic hypoparathyroidism in adults

**Figure 3:**
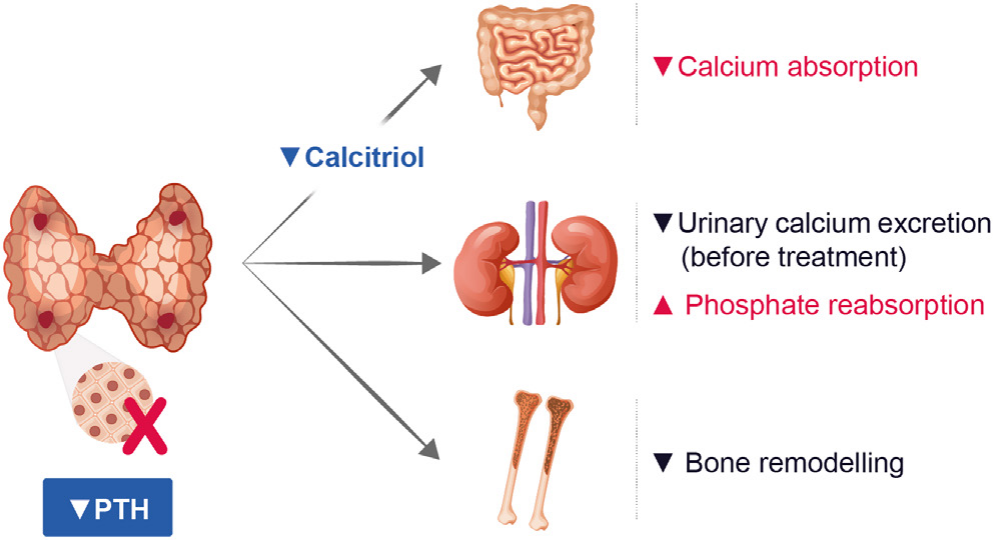
Pathophysiology of hypoparathyroidism. PTH, parathyroid hormone.

HypoPT is a rare endocrine disease characterized by low calcium concentrations due to insufficient PTH secretion from the parathyroid glands (2). In adults, the most common cause is previous anterior neck surgery, in approximately 75% of cases (98). Non-surgical causes include genetic, autoimmune, idiopathic causes, or functional HypoPT related to magnesium depletion and account for the remaining 25% of cases (99). Genetic causes can be isolated or occur as a part of genetic syndromes. In all patients with non-surgical HypoPT, detailed diagnostic work-up is mandatory (2). The chronic PTH deficiency results in decreased intestinal calcium absorption, decreased bone resorption, and decreased renal calcium reabsorption in the distal tubule, accompanied by enhanced phosphate reabsorption in the proximal tubule, causing chronic hypocalcemia and hyperphosphatemia. Urinary calcium excretion may vary according to dietary calcium intake and decreased reabsorption, but is usually lower than after conventional treatment has been started. The pathophysiology of HypoPT is summarized in Fig. 3. Symptoms, related to hypocalcemia, vary from paresthesia and muscle cramps to seizures and life-threatening laryngospasm (99).

In the following section, we will focus on prevention and prediction of the post-surgical HypoPT and will discuss the current treatment modalities and goals, as well as the biochemical monitoring and surveillance, by imaging techniques in adult patients with HypoPT.

### Q1 How do we define chronic postsurgical HypoPT?

HypoPT following anterior neck surgery (mostly thyroid and less frequently parathyroid surgery) is suspected in patients with symptomatic or asymptomatic hypocalcemia and low PTH concentrations, or inappropriately normal PTH concentrations despite hypocalcemia. Calcium should be measured preferably as ionized calcium, or as albumin-adjusted calcium concentrations (2). Calcium and PTH should be assessed repeatedly when the diagnosis is uncertain. The definition of HypoPT differs widely in the medical literature (100, 101). However, to compare the incidence of chronic HypoPT in different countries and among different centers, agreement on diagnostic criteria is essential. We recommend differentiating between transient and chronic post-surgical HypoPT. Chronic post-surgical HypoPT should be diagnosed at least 6 months after the anterior neck surgery, if active vitamin D analogs are still necessary to maintain low normal calcium concentrations. Occasionally, the recovery of the parathyroid tissue may occur later than 6 months. We further recommend confirming the chronicity of post-surgical HypoPT by carefully tapering the treatment in order to avoid misclassification of patients with recovered endogenous parathyroid function.

### Q2 How can postoperative hypoparathyroidism be prevented?

The rate of post-surgical hypoparathyroidism is an internationally accepted outcome variable and an important quality control indicator for endocrine neck surgery (90, 102). Parathyroid preservation with intact gland perfusion and sufficient postoperative function is paramount in endocrine neck surgery. Surgeons performing endocrine neck surgery should be aware of the, sometimes, dramatic impact of permanent HypoPT and the individual risk factors for developing parathyroid dysfunction (Table 3).

**Table 3:** Patients at risk for post-surgical HypoPT and risk mitigation.

Topic	Comment	References
General	Higher rates in • Thyroid cancer with central lymph node dissection • Graves’ disease Risk mitigation • Awareness • Preoperative screening of parathyroid function • Vitamin D status	102, 118, 119
Comorbidities	Obesity Gastrointestinal malabsorption, for example, post gastric bypass, severe IBDRisk mitigation • Awareness	118
Primary exploration vs repeated surgery	Higher risk in • case of repeated surgeryRisk mitigation • critical assessment for additional/repeated surgeries	102
Combined thyroid and parathyroid surgery	Thyroid and parathyroid disease often co-exist but unnoticed if not specifically evaluated Risk mitigation • preoperative evaluation for elective thyroid surgery must include parathyroid function (and vice versa)	102
Total thyroidectomy vs less extensive surgery	Risk bilateral surgery > risk unilateral surgery Risk mitigation • individualized surgical strategy	120
Bilateral cervical exploration in parathyroid disease	Risk bilateral exploration > focused PTX Risk mitigation • preoperative localization (ultrasound and nuclear medicine techniques) • exclusion of FHH (no surgery, see Q1, Primary Hyperparathyroidism)	121

FHH, familial hypocalciuric hypercalcemia; HypoPT, chronic hypoparathyroidism; PHPT, primary hyperparathyroidism; PTX, parathyroidectomy.

Volume and expertise of the center and the individual surgeon influence the rate of postoperative HypoPT significantly (75, 103). Anatomical expertise, early visualization of the parathyroid glands with surgical loupes, meticulous surgical technique, and operative strategies personalized to each patient minimize risk and ensure a favorable outcome. The value of autotransplantation of devascularized or intracapsular parathyroids remains controversial (104). Intraoperative fluorescent imaging of parathyroid glands, as an additional method of visualization and marker of gland viability, may help to reduce postoperative complication rate (105, 106).

**Figure 4:**
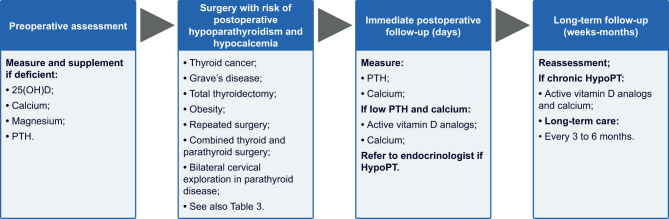
Perioperative management of patients at risk of postoperative hypoparathyroidism. 25(OH)D, 25-hydr oxyvitamin D; HypoPT, chronic hypoparathyroidism; PTH, parathyroid hormone.

We further recommend measuring calcium, magnesium, 25-hydroxyvitamin D (25(OH)D), and PTH in advance of any anterior neck surgery and that vitamin D and magnesium deficiency should be treated. The day following surgery, PTH and ionized calcium should be measured to identify patients at risk of developing HypoPT (Fig. 4).

As stated in 'Q3, Chronic hypoparathyroidism in adults', postoperative PTH monitoring is an integral part of surgical management and allows for the early identification of patients at risk of postsurgical PTH deficiency. Keeping the patient symptom-free and allowing risk-free discharge are the primary goal in the early postoperative phase. Surgeons should initiate interdisciplinary care early for all patients at risk for transient or permanent HypoPT. When immediate postsurgical hypoparathyroidism is diagnosed, it is important to ensure close follow-up in collaboration between the endocrinologist and the general practitioner.

Several important questions remain unanswered and require further research: is parathyroid ‘splinting’ helpful? The concept of parathyroid splinting refers to the hypothesis that high-normal postoperative calcium levels are putting the injured parathyroid parenchyma at rest after thyroidectomy thereby improving long-term parathyroid function (107). Is the identification of the parathyroids beneficial? What is the value of parathyroid tissue cryopreservation? How to estimate how much parathyroid tissue has been removed or damaged? (see ‘How can parathyroid gland injury be predicted?, Q9, Chronic hypoparathyroidism in adults’).

### Q3 How can parathyroid gland injury be predicted?

The prediction of HypoPT from PTH levels following thyroid or neck surgery has to be distinguished from intraoperative PTH measurement in parathyroid surgery. Also with respect to the need to make decisions quickly postsurgically, the assay used for intraoperative measurements provides a rapid result, whereas the routine intact PTH assay may not be appropriate for fast decision-making. With regard to the subsequent threshold levels, different reference ranges dependent on the assay and institution have to be taken into account*.* A very low PTH level on the first postsurgical day is associated with HypoPT (108). Thresholds of PTH concentrations associated with the development of chronic HypoPT have been suggested to be <5.5 pg/mL (<0.58 pmol/L) (109, 110) On the other hand, PTH concentrations on the first post-surgical day >10 pg/mL (>1.06 pmol/L) seem to predict normal parathyroid function 6 months following surgery (111, 112). Another approach is to calculate the ratio between pre- and post-surgical PTH concentrations: a decrease >70% in the normal PTH value is associated with chronic HypoPT (113, 114, 115, 116). Postsurgical PTH measurement has a higher specificity and predictive value compared to calcium measurement (111, 117). Thus, the earliest opportunity to predict hypoPT reliably is through the measurement of serum PTH level at either the conclusion of or immediately following surgery (118). The optimal timing of post-surgical assessment of PTH and the appropriate interpretation of the PTH kinetics remain controversial, as described above.

### Q4 What is the management of acute hypocalcemia following anterior neck surgery?

So far, there is no consensus or guideline on when to initiate postsurgical hypocalcemia with active vitamin D analogs and calcium supplements, or PTH substitution. In case of acute severe hypocalcemic symptoms, such as carpal or pedal spasm or even seizures and laryngospasm, i.v. calcium supplementation has to be initiated (2, 122). The emergency treatment of acute symptomatic hypocalcemia consists of i.v. administration of 200–300 mg (5–7.5 mmol) of elemental calcium by slow i.v. injection, that is, 2–3 ampules of 10 mL of 10% calcium gluconate (N.B. 10 mL of 10% calcium gluconate contains 93 mg/2.3 mmol of elemental calcium) followed by continuous i.v. calcium administration, at a rate of 0.13–0.75 mmol/kg/h (0.5–3 mg/kg/h) in 5% glucose under cardiac monitoring. Milder post-surgical hypocalcemia is treated with oral calcium supplements and active vitamin D analogs (alfacalcidol or calcitriol).

Perioperative management of patients at risk of postoperative HypoPT is illustrated in Fig. 4.

### Q5 What is the first-line treatment of chronic HypoPT?

Chronic HypoPT is conventionally treated with active vitamin D analogs and calcium supplements. As PTH drives 1α-hydroxylation of vitamin D in the proximal tubule, the treatment is based on 1α-hydroxylated analogs of vitamin D. In adults, 0.5–2 μg/day of alfacalcidol (e.g. Un-Alfa®, Etalpha®) once a day or 0.5–1.0 μg/day of calcitriol (e.g. Rocaltrol®) taken twice a day usually allows to obtain acceptable calcium levels (2). During adolescence, the doses of active vitamin D analogs and calcium supplements can be higher because of growth requirements.

Calcium supplements are only needed if dietary calcium is insufficient. They are prescribed in order to increase oral calcium intake, while, when taken with meals, they also act as a phosphate binder. Calcium carbonate, containing 40% of elemental calcium, is most widely used, but calcium citrate, calcium gluconate, or calcium acetate may be preferred in patients taking proton pump inhibitors or suffering from atrophic gastritis (99). An adequate vitamin D status with a serum concentration of 25(OH)D above 20 ng/mL (50 nmol/L) should be ensured (2).

The goal of ‘conventional’ treatment of HypoPT is to maintain calcium concentrations in the lower part or slightly below the lower limit of the reference range with patients being free of symptoms or signs of hypocalcemia (2). No data exist on optimal calcium concentrations during the treatment of HypoPT. Normalization of calcium concentrations very frequently leads to an increase in calciuria, facilitated by the lack of PTH-driven reabsorption of calcium in the distal convoluted and connecting tubule, with the risk of nephrolithiasis and nephrocalcinosis (see ‘Q10, Chronic hypoparathyroidism in adults’). Some patients may, however, need higher calcium concentrations to be symptom-free.

The management of severe episodes of acute symptomatic hypocalcemia defined by calcium concentrations <1.8 mmol/L (<7.21 mg/dL) (or ionized calcium < 0.9 mmol/L (<3.61 mg/dL)), clinical symptoms, or ECG signs, requires emergency treatment with i.v. calcium gluconate and ECG monitoring (99). For emergency treatment of hypocalcemia, see ‘Q4, Chronic hypoparathyroidism in adults’. Episodes of acute hypercalcemia related to overtreatment should be treated rapidly with i.v. fluids, due to the danger of renal impairment following phases of hypercalcemia (123).

### Q6 In which patients should second-line therapies be considered?

The conventional treatment by oral calcium supplements and active vitamin D analogs does not always make it possible to obtain an acceptable clinical and biochemical control of HypoPT, since these treatments do not replace the functions of PTH. In addition, this treatment can lead to short and long-term complications, including hypercalcemia or increased urinary calcium excretion, resulting in nephrocalcinosis or kidney stone formation. The ESE clinical guidelines provide useful treatment goals but do not explicitly recommend when to switch to substitution with the lacking hormone – second-line treatments (2). Different groups and guidelines have suggested that second-line treatment (hormone substitution) could be considered in patients (122):

- with inadequate control of the calcium concentrations;
- in whom calcium supplementation exceeds 2.5 g of elemental calcium/daily or large amounts of active vitamin D analogs are required to control calcium levels or symptoms;
- with hypercalciuria, renal stones, nephrocalcinosis, or reduced renal function;
- with hyperphosphatemia and/or increased calcium–phosphate product;
- with gastrointestinal tract disorder associated with malabsorption;
- with significantly reduced quality of life (QoL).

However, as the clinical benefits and efficacy so far remain unknown, more clinical research is warranted to further define patients who were not adequately controlled by conventional treatment, and which patients would benefit from the second-line treatments.

### Q7 What are the treatment options for chronic HypoPT refractory to conventional treatment?

Two principal therapeutic options may be considered in patients insufficiently controlled by conventional treatment:

Thiazide diuretics (in association with a low salt diet) that reduce urinary calcium excretion and should be considered in patients with hypercalciuria (124). Thiazides are orally administered and are inexpensive. However, they cause a renal loss of sodium, chloride, potassium, and magnesium. Potassium concentrations should be regularly controlled when using thiazides. It should be noted that the clinical benefit of thiazides in HypoPT has so far not been documented.

Substituting the PTH deficiency by s.c. administration of recombinant human PTH (rhPTH) represents another strategy. PTH 1–84 in a daily dose of 25–100 μg has been approved by the U.S. Food and Drug Administration (FDA) and conditionally by the European Medicines Agency (EMA), as an adjunct to conventional treatment for the management of adult patients with HypoPT refractory to conventional treatment (120). However, it is not available in all European countries. Hence, teriparatide, a PTH 1–34 fragment, administered once or twice daily has been used and has proven its efficacy in maintaining normal calcium levels in such patients (125, 126). However, data on reduction of hypercalciuria, preservation of renal function, and bone metabolism with PTH substitution are limited and warrant further investigations (127). More long-acting PTH analogs are currently under development (128, 129).

### Q8 How to evaluate patient’s symptoms and QoL?

Patient-reported outcome measures (PROM) are standardized, validated questionnaires that patients complete by themselves to measure perception symptoms, functional status, and wellbeing (130, 131). In endocrinology, several disease-specific PROMs have been established (e.g. AcroQol, pituitary disease LBNQ-pituitary) (132, 133). QoL is reduced in HypoPT assessed by generic tools as SF-36 (134). The current ESE guidelines do not address PROM (2). More recently, three disease-specific PROMs for HypoPT have been developed, the Hypoparathyroid Patient Questionnaire (HPQ 28, 28 items/questions), the HypoPT Symptom Diary (HSD, 13 items/questions), and the Hypoparathyroidism Patient Experience Scale Symptom (HPES-Symptom, 17 items/questions), and Hypoparathyroidism Patient Experience Scale-Impact (HPES-Impact, 26 items/questions) (135, 136, 137, 138). The HPQ 28 and the HPES have been validated, while the shorter HSD so far lacks validation.

In contrast to most methods of biochemical monitoring, PROM may reflect calcium metabolism better over time, include the patients’ perspective, and support shared decision-making. Furthermore, a semi-quantitative approach to the patient’s perception may improve the traditionally qualitative character of most patient consultations (131).

We agree upon the importance of a standardized assessment of symptoms and QoL. However, awareness of unintended effects on the patients and patient communication must be considered. The repeated reminders of symptoms and discomfort may have a negative effect on the patients’ overall wellbeing (139). The present PROMs for HypoPT need a broader validation, both as research and clinical tools. A meaningful, efficient, and successful clinical implementation requires the development of user-friendly electronic applications for the patients and health care providers and broad validation.

### Q9 Which biochemical parameters should be monitored to adjust treatment?

The principal parameter to monitor in patients with HypoPT is serum calcium concentration. A discussion is ongoing about which calcium to measure: free-ionized, total, or albumin-adjusted calcium concentrations. One recent study (140) suggests that adjustment formulas misclassify patients and add no value as compared to total calcium concentrations, while new equations are still being developed (141). At steady state, we recommend measuring calcium concentrations every 3–6 months. Calcium should be monitored more often in patients requiring dose adjustment of calcium or vitamin D treatment or during intercurrent illness, pregnancy, lactation, etc (2) Table 4.

**Table 4:** Biochemical parameters in hypoparathyroidism.

Lab test	Looking for	Interval	Comments
Calcium	Hypocalcemia and hypercalcemia	At every check, every 6 months at steady state	Ionized calcium is preferable If not available, total calcium (and albumin-corrected) is acceptable Timing of assessment is dependent on previous/daily calcium intake by food or supplements, as well as treatment Calcium levels should be assessed several days after changes in active vitamin D analog doses or PTH doses to detect iatrogenic hypercalcemia
PTH		Only for diagnosis	Not required for follow-up in chronic HypoPT Should be assessed to detect recovery in transient post-surgical hypoparathyroidism (6–12 months after the surgery)
Phosphate	Hyperphosphatemia	At every check	Hyperphosphatemia can be related to high dietary phosphate intake (soft drinks, products with preservatives, acidifier, and flavor enhancer) Hyperphosphatemia is associated with higher risk of infections and with increased mortality (142)
Calcium–phosphate product			Should be calculated Associated with brain calcifications and reduced QoL in some (144, 145) but not all studies (3)
Kidney function	Renal insufficiency	At every check	To detect decline in renal function Advise patients to be careful with nephrotoxic medications and with dehydration (146)
25(OH)D	Vitamin D deficiency		Often high vitamin D doses needed, especially under PTH replacement therapy 25(OH)D recommended goal: >30 ng/mL and <50 ng/mL (>75 nmol/L and <125 nmol/L) (expert opinion (2))
Calciuria	Hypercalciuria	Every 6–12 months	24 -h calcium excretion is reliable and spot easier to obtain
Urinary stone profile		As clinically indicated	Sodium, urea, citrate, oxalate, pH, osmolarity, urate excretions, and others
Magnesium	Hypomagnesemia	Yearly or as clinically indicated	Serum magnesium does not reflect intracellular levels well and magnesium depletion is possible with normal values Hypomagnesemia reduces response to PTH and may cause hypokalemia
Thyroid status		At every check	In patients with thyroid replacement therapy

25(OH)D, 25-hydroxyvitamin D; HypoPT, chronic hypoparathyroidism; PTH, parathyroid hormone; rhPTH, recombinant human parathyroid hormone.

Serum phosphate concentrations and the calcium–phosphate product should be monitored. Some observational studies have shown a strong association with any kidney disease, nephrocalcinosis, the development of brain calcification, and QoL (142, 143, 144, 145). However, it is still unclear whether decreasing levels of phosphate/calcium–phosphate product would improve renal outcomes, Table 4.

Kidney function should be monitored (through a measure of creatinine) at least every year in patients with HypoPT (146), but it is unclear whether it is possible to prevent a renal function decline. So far, no clinical interventional study has shown improvement of renal function by any HypoPT treatment. A study suggested a benefit of rhPTH(1–84) treatment as compared to the standard of care, but groups were too heterogeneous to draw firm conclusions (147), Table 4.

We recommend that phosphate and creatinine concentrations should be measured every 3–6 months, depending on disease control and stability of the patient. Serum magnesium concentrations should be measured at least annually, but more frequently if low. Biochemical markers of bone turnover could be useful in assessing the effect of PTH replacement therapy on bone homeostasis, Table 4.

Urinary calcium excretion measurement is often neglected during follow-up. The assessment of urinary calcium excretion identifies patients at risk of developing kidney stones and/or nephrocalcinosis and decline of renal function. Even if recent studies did not find a clear correlation between kidney stones and hypercalciuria (148), or between urinary biochemical parameters and kidney outcomes (146), patients with HypoPT should be considered at a high risk of developing nephrolithiasis, nephrocalcinosis (149), as well as chronic kidney disease (146), Table 4.

Current guidelines suggest monitoring 24-h urine calcium excretion in all HypoPT patients without clarifying its frequency (2) or recommend an evaluation every 6–12 months (4, 122). This evaluation usually includes 24-h urine creatinine excretion to ensure a complete 24-h collection. Some debate still exists on the value of measuring calcium-to-creatinine ratio rather than a 24-h urine excretion. Assessment of urinary biochemical profile, including the excretion of sodium, urea, citrate, oxalate, urate, and other analyses like pH and osmolarity (99), is of importance, in particular in patients with history of nephrolithiasis, Table 4.

### Q10 Which imaging techniques are useful to monitor treatment or to evaluate tissue complications of chronic HypoPT?

The ESE guideline on the management of HypoPT did not comment on the approach of patient surveillance by imaging during follow-up (2). Therefore, questions remain unanswered on the optimal imaging approach for baseline evaluation and long-term surveillance of patients with chronic HypoPT. Clinicians should be aware of common adverse end-organ manifestations (e.g. renal or CNS calcification) and organize imaging to detect these accordingly, depending on the clinical situation (Table 5).

**Table 5:** Imaging in hypoparathyroidism.

Organ	Looking for	Interval	Comments
Kidney	Nephrolithiasis, nephrocalcinosis	At diagnosis;As clinically indicated;Every 5 years	Ultrasound + no radiation - highly operator-dependent Non-contrast renal CT + accurate - accumulation of radiation exposure *Sensitivity of CT vsultrasound in nephrocalcinosis detection uncertain*
Brain	Intracerebral calcifications	As clinically indicated	Non-contrast CT MRI (only special MRIs usable for this assessment). *Sensitivity of even specialized MRI for detection of calcifications uncertain*
Bone	Changes in bone density/quality, vertebral fx	As clinically indicated	DXA + cheap + low radiation + Vertebral fracture assessment (VFA) X-ray spine and VFA + detection of unknown vertebral fracture
Eyes	Cataract	At diagnosis;As clinically indicated	Ophthalmologist check in non-surgical patients

CT, computed tomography; DXA, dual-energy X-ray absorptiometry; HypoPT, chronic hypoparathyroidism; MRI, magnetic resonance imaging; Fx, fractures.

#### Renal and urinary tract imaging

Regular renal imaging (every 1–2 years) is required to detect calcification or stones at an early stage in asymptomatic individuals. Renal ultrasound has advantages in terms of wide availability, acceptability, and safety. In patients with prior stones, involvement of a urologist is indicated with interval scans depending on the determination of individual risk. CT scan of the renal tract has higher sensitivity and we would advocate its use in symptomatic individuals. However, it should be indicated with caution with regards to regular long-term surveillance imaging to minimize risk from radiation dosing. Clinical parameters such as urinary calcium excretion and dynamics of renal function should also be used to stratify patient risk of stone formation and determine timing of scans.

#### CNS effects

Basal ganglia calcification and other brain tissue calcifications are commonly associated with chronic HypoPT, particularly in patients with genetic and autoimmune disease, although studies of postsurgical cases had limited duration of follow-up (144, 150). There is a wide spectrum of CNS manifestations of this condition, including movement disorders, seizures, and neuropsychiatric symptoms. A careful clinical assessment including brain imaging is recommended in patients with these manifestations. CT is commonly used to detect basal ganglia calcification; however, it is associated with exposure to ionizing radiation (151). Standard MRI has low sensitivity for the detection of calcification, whereas susceptibility-weighted MRI (SW-MRI) is validated for this indication, but CT seems to be more sensitive. Prospective assessment of the natural history of basal ganglia calcification in HypoPT with clinical correlation would be helpful to develop protocols in this area. Data for guidance on mode and frequency of corresponding imaging surveillance in asymptomatic patients are lacking.

#### Monitoring of bone mineral density

BMD tends to be increased in patients with chronic HypoPT with evidence of reduced bone turnover (152, 153), but the clinical implication is unclear. In preexisting conditions affecting the bone (PHPT, osteoporosis, and hyperthyroidism), BMD measurement should be considered. Assessment of BMD and fracture risk, in line with standard guidelines, follows a pragmatic approach. Prospective data on meaningful clinical bone outcomes, such as fracture rates, are needed (154). We do agree on the importance of biomarkers for bone turnover in these patients; however, it is questionable if BMD measurement is helpful in monitoring. DXA could be considered in patients receiving long-term PTH replacement therapy with increase bone turnover markers (127, 155).

## Parathyroid disorders during preconception, pregnancy, and lactation

**Figure 5:**
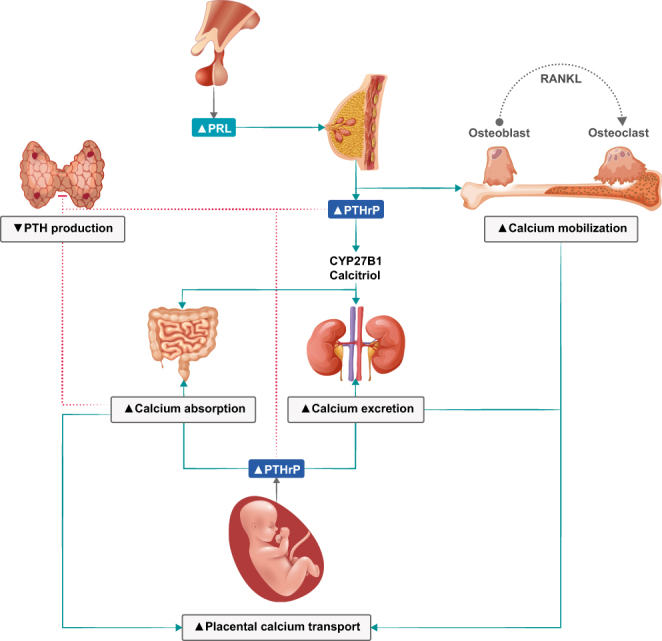
Overview of calcium homeostasis and calciotropic hormones in pregnancy. Parathyroid hormone-related protein (PTHrP) production in the placenta will gradually decrease endogenous parathyroid hormone (PTH) secretion (4, 5). PRL, prolactin; RANKL, receptor activator of nuclear factor kappa-Β ligand.

Changes in bone and mineral metabolism during pregnancy and lactation occur to ensure an adequate mineral supply to meet the growing needs of the fetal skeleton and the newborns while preserving maternal health (Fig. 5) (4, 5, 8, 156, 157, 158). During pregnancy, the plasma volume expansion with subsequently reduced albumin levels causes a decrease in total calcium concentrations. Ionized (free) and albumin-adjusted calcium remain unchanged in pregnant women with normal parathyroid function and should therefore be used for clinical decisions on calcium and parathyroid-related disorders during pregnancy.

Fetal mineral demands are mainly met by approximately doubling the intestinal absorption of calcium, phosphate, and magnesium, that is, at least in part, a consequence of increases in 1,25(OH)_2_D and estrogen. This and glomerular hyper-filtration lead to increased postprandial and 24-h urine calcium excretion with normal fasting urine calcium excretion (i.e. absorptive hypercalciuria) (159). Required minerals are actively transported by the placenta and circulate at higher concentrations in the fetus as compared to the mother (157). This facilitates mineralization of the fetal skeleton before birth, with 80% of mineral accretion occurring in the third trimester (157). Despite this mineral supply, pregnancy itself usually does not alter maternal BMD if the maternal calcium intake is adequate or result in only a modest 1–4% decrease in BMD (158). During lactation, mineral supply to breast milk mainly derives from the maternal skeleton that is exposed to increased bone resorption and osteocytic osteolysis (156). This translates into a significant decrease of about 5–10% in lumbar spine BMD after 6 months of exclusively nursing a singleton, with about half of this effect at the hip and radius (156, 160). Six to 12 months after weaning, the maternal BMD reaches its pre-pregnancy values or may even exceed them (156). Therefore, and although the literature on this issue is not fully consistent, parity and lactation itself do usually not increase the long-term risk for osteoporosis and fractures, but might even confer some protection (8, 161).

During pregnancy, maternal PTH, which does not cross the placenta, is usually suppressed to the lower normal range or even below, while 25(OH)D remains materially unchanged and 1,25(OH)_2_D increases two- to three-fold (158). Parathyroid hormone-related peptide (PTHrP) that is virtually not detected in non-pregnant women increases steadily during pregnancy and appears to be mainly produced by breast tissue and placenta (5, 158). The precise physiological role of PTHrP is still not fully clarified but has been shown to exert PTH-like effects. Excess PTHrP production may cause hypercalcemia, a disease termed ‘pseudohyperparathyroidism’ (5, 158). During lactation, PTHrP release is stimulated via suckling and high prolactin, as exemplified by the successful treatment of pseudohyperparathyroidism with prolactin-suppressing dopamine agonists (8, 162). After delivery and during lactation, 1,25(OH)_2_D concentrations normalize, while inconsistent data have been reported on PTH concentrations (8).

No RCTs are available to guide management decisions for parathyroid disorders during pregnancy and lactation. Accordingly, all recommendations and suggestions are based on (limited) evidence from observational studies and personal experience.

In general, we consider it as a reasonable approach to measure calcium concentrations (preferably albumin corrected and/or ionized calcium) at least once as part of otherwise indicated routine screening programs or visits during preconception and early pregnancy and consult an endocrinologist in case of calcium levels outside the reference ranges.

### Management of patients with PHPT in relation to pregnancy?

**Figure 6:**
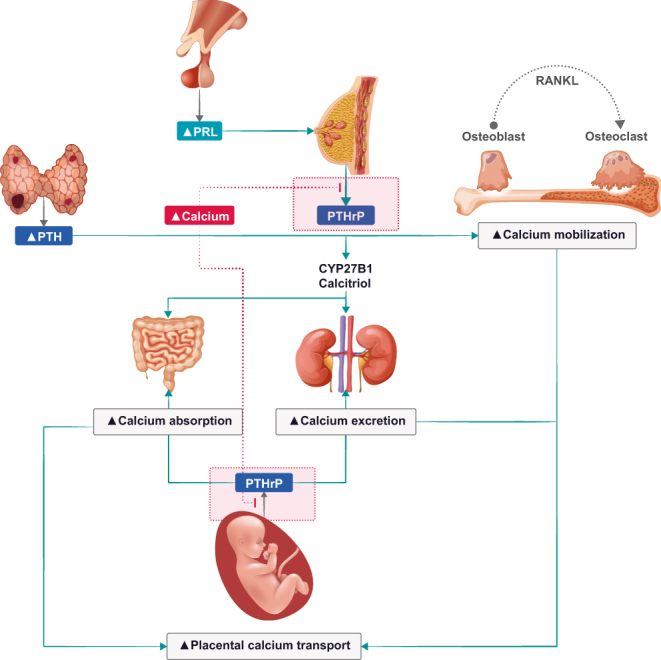
Overview of calcium homeostasis in primary hyperparathyroidism during pregnancy. Parathyroid hormone (PTH) and parathyroid hormone-related protein (PTHrP) will counterbalance the equilibrium hypercalcemia (5, 25, 158). PRL, prolactin; RANKL, receptor activator of nuclear factor kappa-Β ligand.

PHPT during pregnancy is relatively rare, accounting for roughly 1% of the total PHPT patients (163, 164, 165). PHPT diagnosed during pregnancy requires consideration of alterations in PTH concentrations and associated changes in mineral metabolism (Fig. 6) (163, 164, 165, 166, 167, 168, 169, 170, 171, 172). Distinguishing PHPT from FHH in pregnancy is challenging in view of absorptive hypercalciuria, resulting in a significant increase of the CCCR (173, 174). Therefore, laboratory values of first-degree relatives and of the patient before pregnancy, as well as genetic testing, are of particular importance in this setting (173, 174).

In case reports and case series, maternal clinical symptoms of PHPT during pregnancy vary considerably and resemble frequent complaints during normal pregnancy, such as malaise, nausea, vomiting, fatigue, polyuria, or muscle weakness. Polyhydramnios may develop due to osmotic polyuria of the fetus (171). The vast majority of investigations on gestational PHPT report on significantly increased risk of fetal/neonatal and maternal complications (163, 164, 165, 166, 167, 168, 169, 170, 171, 172), which might reflect publication bias. In general, calcium levels are usually only slightly elevated and relatively stable, and many cases of mild PHPT go undiagnosed in pregnancy with no particular increase in maternal or fetal health risk (159).

Nevertheless, PHPT during pregnancy has been reported to increase the risk of miscarriages, premature birth, intrauterine growth retardation and maternal nephrolithiasis, pancreatitis, and preeclampsia (164, 165, 166, 167, 168, 169, 172, 173). There seems to be a strong positive correlation between calcium concentrations and pregnancy complications, with the risk being particularly increased if total calcium levels are >2.85 mmol/L (>11.42 mg/dL). By contrast, there is still some controversy whether mild hypercalcemia is associated with adverse pregnancy outcomes (163, 166, 169).

#### Q1 What preconception advice should be given to women with PHPT?

We recommend that pregnancy, if possible, should be deferred until curative surgery for PHPT has been performed and calcium levels have been normalized.

#### Q2 How to treat PHPT during pregnancy?

International guidelines recommend PTX in patients below the age of 50 years (28). Therefore, the main question is the timing and mode of surgery, while conservative management should only be regarded as temporary until surgery. Conservative treatment options primarily include oral and i.v. rehydration and cinacalcet for severe hypercalcemia (5, 8). Cinacalcet has been used in several pregnant women without significant safety concerns, although it crosses the placenta, and safety evidences are still considered insufficient for official approval. Safety concerns argue against the use of bisphosphonates that also cross the placenta (159). Calcitonin does not cross the placenta, but its efficacy to control hypercalcemia declines after a few days due to tachyphylaxis. Importantly, a systematic review of observational studies including 382 women with gestational PHPT, of whom 108 underwent PTX during pregnancy, reported a significantly lower infant complication rate for surgery vs medical therapy (9.1% vs 38.9%), with similar results when restricting the analyses to asymptomatic cases (168). Adverse outcomes were less likely when surgery was performed in the second vs the third trimester (4.5% vs 21.1%). Accordingly, we recommend that pregnant women with PHPT and an albumin-adjusted total calcium level >2.85 mmol/L (>11.42 mg/dL) and/or >0.25 mmol/L (>1 mg/dL) above ULN and/or an ionized calcium > 1.45 mmol/L (>5.81 mg/dL) should undergo PTX in the second trimester. For pregnant PHPT women with calcium levels below the above-mentioned cut-offs, an individual shared decision should be made regarding the therapeutic options. However, PTX may be considered in selected cases, but factors, such as only mild hypercalcemia and/or doubts regarding the correct diagnosis of PHPT might justify a conservative approach. For surveillance, we consider it as a reasonable approach to monitor calcium levels and eGFR approximately every 4 weeks in pregnant women with PHPT, and even more frequently (e.g. all 1–2 weeks) after changes in the medical treatment of PHPT. In case of PTX, perioperative laboratory measurements and calcium plus vitamin D supplementation are recommended, as in non-pregnant patients (Table 6).

**Table 6:** Summary of recommendations for PHPT and HypoPT during preconception, pregnancy, and lactation.

	PHPT	HypoPT
Mother (preconception)	Pregnancy should be avoided until curative surgery has been performed and calcium concentrations are normalized	Counselling regarding frequent surveillance and potential changes in vitamin D and calcium requirements during pregnancy Most mothers will have a healthy baby
Mother (pregnancy)	Surgery is advised, preferentially in the second trimester, and especially if albumin-adjusted calcium is >2.85 mmol/L (>11.42 mg/dL) and/or >0,25 mmol/L (>1 mg/dL) ULN and/or ionized calcium is >1.45 mmol/L (>5.81 mg/dL) Surveillance every 4 weeks	Calcium and vitamin D supplements, as well as active vitamin D treatment can be used Aim for ionized and/or albumin-adjusted calcium levels in the lower end of the reference range. Surveillance every 3–4 weeks; each week during the last month of pregnancy
Mother (lactation)	Surveillance every 4–8 weeks Surgery a few weeks after delivery	Surveillance weekly within the first month after birth and then every 4 weeks
Newborns	Measure ionized calcium every second day until about 1–2 weeks of life In case of hypocalcemia, consider active vitamin D treatment	Measure ionized calcium every second day during the first week of life

HypoPT, chronic hypoparathyroidism; PHPT, primary hyperparathyroidism; ULN, upper limit of normal.

#### Q3 What is the preferred surgical approach for PTX during pregnancy?

In sporadic PHPT, minimally invasive PTX in combination with intraoperative PTH monitoring is preferred. Bilateral neck exploration may be needed in hereditary forms of PHPT (i.e. MEN1, MEN2A, MEN4, hyperparathyroidism-jaw tumor syndrome, and familial isolated hyperparathyroidism) and other causes of parathyroid hyperplasia with potential indication for surgery, such as lithium intake (175).

#### Q4 Which parathyroid imaging modalities should be used in pregnant women with PHPT and indication for surgery?

Preoperative localization of abnormal parathyroid glands is crucial for the success of minimally invasive PTX, and concordance of two imaging methods may result in very high positive predictive values for correct localization of adenoma(s) (176). Avoiding radiation exposure in pregnancy is critical for guiding decisions on the choice of imaging methods for PHPT. Ultrasound and 4D-dynamic contrast-enhanced MRI lack ionizing radiation, but if needed, also ^99m^Tc-methoxyisobutylisonitrile (99mTc-MIBI) scans, sestamibi single-photon emission CT (SPECT/CT), 18F-Fluorocholine PET/CT, or methionine PET/CT are acceptable choices after careful consideration of potential risks and benefits.

The fetal exposure by a 99mTc-MIBI scan is lower than the exposure associated with fetal harm (177). 18F-Fluorocholine PET/CT is even considered to have a lower overall (maternal) radiation dose than 99mTc-MIBI SPECT/CT, but the fetal radiation exposure by 18F-Fluorocholine PET/CT is not exactly known and might even be higher due to increased fetal tracer uptake (176). 4D-CT has the highest radiation dose and should be avoided during pregnancy (176). To provide rough guidance for pregnant women with PHPT and an indication for surgery, we consider it reasonable to aim for two different preoperative imaging methods, preferably ultrasonography in combination with either 99mTc-MIBI scan or 4D-dynamic contrast-enhanced MRI. Other imaging methods or ultrasonography without additional imaging may be considered for selected cases and taking into account the local expertise and the individual risks and benefits. However, any imaging method with radiation exposure should only be performed if considered to have a favorable benefit to risk ratio for the individual pregnant woman.

#### Q5 What are important considerations for the postpartum and lactation period for the mothers with PHPT and their newborns?

In pregnant women with PHPT, it should be considered that fetal PTH is likely suppressed due to elevated maternal calcium levels, but immediately after birth, the calcium transfer *via* the placenta is abruptly stopped. Consequently, there is an increased risk of hypocalcemia and hypocalcemic seizures (clonic movements) in the newborns and of maternal hypercalcemic crisis in the postpartum period. During lactation, we consider it reasonable to measure maternal calcium levels and eGFR approximately every 4–8 weeks starting within the first week after delivery (Table 6). PTX should be performed a few weeks after delivery, when the mother has fully recovered. With respect to medical treatment, cinacalcet is excreted in the milk of lactating rats with high milk to plasma ratio. Therefore, a careful benefit/risk assessment decision should be made to discontinue either breastfeeding or treatment with cinacalcet in lactating women who require this treatment for PHPT (23/01/2020 Mimpara – EMEA/H/C/000570 – II/0065). We recommend close clinical monitoring for the newborns of mothers with PHPT as neonatal hypocalcemia may occur within the first 2–3 weeks of life. We suggest to measure ionized calcium in the newborns at least every 2 days starting on day 2, and continuing this, depending on disease severity, until about 1–2 weeks of life, with usually no further testing in case of mild disease with normal neonatal calcium levels within the first week of life. Consider active vitamin D treatment (e.g. alfacalcidol or calcitriol) in case of neonatal hypocalcemia that usually occurs around day 2 or 3. Regular native vitamin D supplementation (usually 400 international units cholecalciferol (vitamin D3) per day is recommended.

### Management of HypoPT patients in relation to pregnancy

Management of chronic HypoPT during pregnancy constitutes a challenge, as there are scarce data on this topic. Most published studies are based on case reports or small case series, which suggest that maternal HypoPT is associated with significant maternal and fetal morbidity, including fetal loss and preterm delivery (178, 179, 180, 181). Importantly, the by far largest study on this topic, a recently published register study from Sweden of 97 pregnant women with chronic HypoPT and 1030 pregnant age-matched controls, showed a significantly higher prevalence of diabetes mellitus and chronic kidney disease in chronic HypoPT compared with controls (182). In multivariate adjusted analyses, chronic HypoPT vs controls was associated with higher risk of induction of labor and slightly lower birth weight, but no other adverse pregnancy outcomes (182).

**Figure 7:**
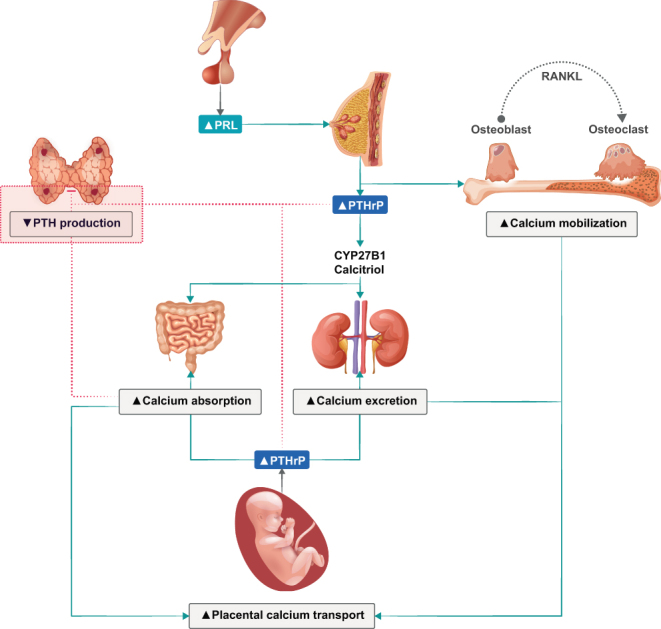
Overview of calcium homeostasis in chronic hypoparathyroidism during pregnancy. PTH, parathyroid hormone; PTHrP, parathyroid hormone-related protein; PRL, prolactin; RANKL, receptor activator of nuclear factor kappa-Β ligand.

Calcium requirements increase during pregnancy. It is important to ensure an adequate intake of calcium and active vitamin D, as maternal hypocalcemic may cause secondary hyperparathyroidism in the fetus with fetal skeletal demineralization (Fig. 7) (183, 184). In contrast, if the mother is hypercalcemic, the fetal parathyroid tissue may become suppressed and the fetus may develop hypocalcemia (179). It is therefore of particular importance to closely monitor women with HypoPT during pregnancy. Recently, recommendations for the management of HypoPT during pregnancy (185) were released, followed by an expert consensus statement (4). Calcium and activated vitamin D supplements were recommended as the mainstay of treatment for HypoPT during pregnancy (Table 6) (4, 185).

#### Q6 What are the treatment targets and surveillance recommendations during pregnancy in women with chronic HypoPT?

The goal of therapy is to achieve an ionized and/or albumin-adjusted calcium level in the lower end of the normal reference range and to avoid both maternal hypo- and hypercalcemia. As it is difficult to predict precise doses of calcium and active vitamin D needed to maintain the concentrations within the recommended range, careful monitoring of calcium, phosphate, eGFR, and magnesium should be performed during pregnancy, that is, every 3–4 weeks, and more frequently (e.g. weekly) within 1 month before giving birth (185). If changes in the dose of calcium and/or active vitamin D are recommended, measurement of calcium should be repeated in 1–2 weeks. It is recommended to maintain 25(OH)D levels in the reference range (Table 6) (185).

#### Q7 What are the required doses of calcium and active vitamin D during pregnancy in women with chronic HypoPT?

The literature describes a wide variation in the required doses of calcium and active vitamin D (alfacalcidiol or calcitriol) to maintain normocalcemia during pregnancy in women with chronic HypoPT (180, 181). Some women require higher doses of active vitamin D, whereas others require lower doses. These dose requirements may also change during the course of pregnancy (180, 181). Individual variations may, among others, reflect variations in dietary intakes, and variations in PTHrP production, and in calcitriol production from the maternal kidneys (Fig. 7) (180). Notably, inadequate calcium intake in the first trimester may contribute to inadequate mineral accrual of the developing fetus.

#### Q8 Is treatment with PTH analogs and hydrochlorothiazide safe during pregnancy?

Adjunctive therapy with PTH analogs has not been evaluated during pregnancy (FDA Category C drugs), and hydrochlorothiazide (FDA Category B drug) should be stopped during pregnancy in the first trimester and should only be used after careful risk evaluation in the second and third trimester (4).

#### Q9 How to manage chronic HypoPT during lactation?

Close monitoring of calcium is recommended during lactation as calcitriol levels normalize (i.e. endogenous calcitriol synthesis decreases after delivery), PTHrP levels are increased, bone resorption increases, and renal calcium reabsorption increases (186). These effects may lower the dose requirements for active vitamin D and calcium supplementation during lactation in women with chronic HypoPT. Therefore, it is recommended to continue monitoring maternal calcium levels weekly within the first month after delivery and then every 4 weeks during lactation (184). Abrupt cessation of breastfeeding can be associated with maternal hypocalcemia.

#### Q10 What are the surveillance and treatment recommendations for newborns of mothers with HypoPT?

Newborns should be monitored clinically and we suggest measuring ionized calcium concentrations every second day for the first week of life (Table 6). Regular native vitamin D supplementation is recommended.

## Main statements and recommendations

Main recommendations from each of the chapters are given in bullet points.

### Primary hyperparathyroidism

#### Q1 How do we differentially diagnose FHH?

- Positive family history is a key feature of FHH, so family testing has a pivotal role.
- Historic calcium values are important to exclude progressive hypercalcemia as in PHPT.
- PTH concentrations > 2-fold above ULN are suggestive of PHPT.
- A CCCR of <0.01 (i.e. an excretion fraction of calcium <1%) is a screening tool for FHH, but the ‘cut-off’ is of limited clinical value due to low diagnostic sensitivity and specificity.
- Genetic testing is recommended for all patients with suspected FHH, as identification of a pathogenic mutation will confirm the diagnosis and differentiate between types of FHH.
- Negative genetic testing does not exclude FHH, and we recommend ongoing follow-up of mutation-negative patients.

#### Q2 What is normocalcemic PHPT?

- Normocalcemic PHPT is a biochemical signature of persistently (>3 months) increased PTH levels in the setting of consistently normal calcium concentrations.
- Normocalcemic PHPT represents a diagnosis of exclusion and can only be considered following a careful evaluation of causes of secondary hyperparathyroidism.

#### Q3 What are the causes of hyperparathyroidism with normal calcium that should be excluded before considering a diagnosis of normocalcemic PHPT?

- Detailed and standardized tests are required before making a positive diagnosis of normocalcemic PHPT, and secondary causes of hyperparathyroidism must be excluded.

#### Q4 What are the manifestations of normocalcemic PHPT and does it progress to hypercalcemic PHPT?

- Normocalcemic PHPT may be an early biochemical manifestation of PHPT.
- There are no clear data on the natural history of normocalcemic PHPT.

#### Q5 What is the definition, prevalence, and causes of recurrent PHPT?

- The most widely accepted definition of recurrent PHPT is a demonstration of hypercalcemia, after a period of 6 months, in patients successfully operated by PTX, and where normocalcemia was previously documented.
- The true prevalence is unknown but estimated to be between 2.5 and 10%.
- The recurrence can be tardive, wherefore long-term follow-up is recommended following successful PTX.

#### Q6 Do we need to act upon persistent elevations of PTH levels, despite normocalcemia?

- PTH should not be routinely measured in normocalcemic individuals following parathyroid surgery.

#### Q7 What is the optimal work-up of patients with recurrent PHPT?

- When evaluating recurrent PHPT, it is mandatory to accurately confirm or refute the diagnosis of PHPT.
- If confirmed, an active search for potential underlying etiologies should be considered.

#### Q8 What is the best surgical approach in patients with recurrent PHPT?

- A thorough preoperative work-up is imperative, and repeat surgery should only be performed in highly experienced centers.
- Depending on the results and etiology, bilateral neck exploration or a focused minimal-invasive PTX should be performed.

#### Q9 What is the risk and prevention of hypoparathyroidism following surgery for recurrent PHPT?

- In the reoperative setting, the risk of transient hypoparathyroidism can be as high as 80%, while the rate of chronic HypoPT is 3–13%.

#### Q10 Why and when should calcium levels be measured after PTX?

- Calcium levels should be measured postoperatively, in parallel to evaluation for symptoms of low calcium concentrations.
- To define cure of PHPT after PTX, normocalcemia should last a minimum of 6 months.

#### Q11 What preoperative advice should be offered to patients awaiting PTX?

- Low 25(OH)D levels should be repleted.
- Patients should stay well-hydrated.
- Hypercalcemic crises require parenteral hydration and may benefit from further medical management; surgery might be prioritized in selected cases after medical stabilization.

#### Q12 What causes hypocalcemia after PTX?

- Postoperative hypocalcemia can be related to hypoparathyroidism (inappropriate low PTH in relation to calcium concentration), accompanied by a high phosphate concentration.
- HBS: Massive transfer of calcium to bone – is characterized by normal or high PTH, low phosphate, and magnesium.

#### Q13 What is optimal follow-up after (successful) PTX?

- Genetic testing in young patients (<30 years) and multiglandular disease at any age.
- Special cases (parathyroid cancer, syndromic forms) should be followed with a personalized plan in a specialized endocrine unit.
- Patients with concomitant osteoporosis are in need of individualized management.

### Chronic hypoparathyroidism in adults

#### Q1 How do we define chronic postsurgical HypoPT?

- Postsurgical HypoPT is suspected in patients with symptomatic or asymptomatic hypocalcemia and low or inappropriately ‘normal’ PTH concentrations.
- Chronic postsurgical HypoPT is diagnosed 6 months after anterior neck surgery, if medication is still necessary to maintain calcium concentrations in the lower normal range.

#### Q2 How can postoperative hypoparathyroidism be prevented?

- We recommend that anterior neck surgery is performed only by experienced surgeons, in particular, in patients at risk of postsurgical hypoparathyroidism.
- When immediate postsurgical hypoparathyroidism is diagnosed, we recommend a close and coordinated follow-up team including the surgeon, the endocrinologist, and the general practitioner.

#### Q3 How can parathyroid gland injury be predicted?

- We recommend early postoperative PTH monitoring (rather than isolated calcium assessments) to identify patients at risk of post-surgical parathyroid deficiency.

#### Q4 What is the management of acute hypocalcemia following anterior neck surgery?

- The emergency treatment of acute symptomatic hypocalcemia (defined by calcium levels <1.8 mmol/L (<7.21 mg/dL), or ionized calcium <0.9 mmol/L (<3.61 mg/dL) with clinical symptoms and/or ECG signs) consists of the i.v. administration of 200–300 mg (5–7.5 mmol) of elemental calcium by slow i.v. injection, that is 2–3 ampules of 10 mL of 10% calcium gluconate.
- Mild postsurgical hypocalcemia should be treated with oral calcium supplements and active vitamin D analogs.

#### Q5 What is the first-line treatment of chronic HypoPT?

- We recommend as first-line treatment of chronic HypoPT active vitamin D analogs (alfacalcidol or calcitriol), combined, when necessary, with oral calcium supplements (mostly calcium carbonate) in divided dosages.
- The goal of treatment is to maintain calcium concentrations in the lower part or slightly below the lower limit of the reference range with patients being free of symptoms. Some patients may, however, need higher calcium concentrations to be symptom-free.

#### Q6 In which patients should second-line therapies be considered?

- The conventional treatment does not fully replace PTH actions and therefore does not permit obtaining acceptable disease control in all patients.
- We recommend considering second-line treatments (hormone substitution) in patients with inadequate calcemic control, with hypercalciuria, renal stones, nephrocalcinosis, or reduced renal function, with hyperphosphataemia and/or increased calcium–phosphate product, with gastrointestinal tract disorder with malabsorption, with excessive doses of calcium supplementation and/or active vitamin D analogs, and with significantly reduced QoL.

#### Q7 What are the treatment options for chronic HypoPT refractory to conventional treatment?

- We recommend adjunction of thiazide diuretics to the conventional therapy to reduce urinary calcium excretion in patients with hypercalciuria.
- Substitution therapy with rhPTH(1–84), or, if unavailable, with rhPTH(1–34), may be considered in patients with hypocalcemia despite optimized conventional therapy, in patients with malabsorption, and with significantly reduced QoL. However, long-term consequences of PTH replacement therapy on the kidney and bone warrant further investigations.

#### Q8 How to evaluate patient’s symptoms and QoL?

- Three disease-specific patient-reported outcome measures are available for HypoPT: the Hypoparathyroid Patient Questionnaire (HPQ 28), the HypoPT Symptom Diary (HSD), and the Hypoparathyroidism Patient Experience Scale Symptom and Impact (HPES-Symptom, HPES-Impact). They represent a valuable tool to evaluate the fluctuations of calcium homeostasis over time but necessitate a broader validation both as research and clinical tools.

#### Q9 Which biochemical parameters should be monitored to adjust treatment?

- We recommend measuring free-ionized or albumin-adjusted calcium concentrations every 3–6 months, and more frequent monitoring in patients requiring treatment adjustment or during intercurrent illness.
- We recommend phosphate monitoring every 3–6 months and strongly suggest calcium–phosphate product calculation at every check.
- We recommend monitoring renal function with creatinine measurement at every check.
- Magnesium concentrations should be measured at least annually and at every check in patients with hypomagnesemia.
- Measuring 24-h urinary calcium and creatinine excretions are important to identify patients at risk of developing kidney stones and/or nephrocalcinosis and should be performed every 6–12 months. Urinary calcium/creatinine ratio calculated from fasting morning spot urine is an alternative.

#### Q10 Which imaging techniques are useful to monitor treatment or to evaluate tissue complications of chronic HypoPT?

- We recommend surveillance by renal imaging every 1–2 years to detect nephrolithiasis or nephrocalcinosis. Repeated renal CT scans should be indicated with caution to minimize risk from radiation dosing.
- We recommend performing brain CT scans to search for basal ganglia or other brain tissue calcifications in patients with neurologic symptoms including movement disorders, seizures, and neuropsychiatric symptoms.
- Whether BMD measurements are helpful in the regular care of patients is questionable.

### Parathyroid disorders in relation to pregnancy

#### Q1 (PHPT) – What preconception advice should be given to women with PHPT?

- If possible, pregnancy should be deferred until curative surgery for PHPT has been performed.

#### Q2 (PHPT) – How to treat PHPT during pregnancy?

- PTX is preferred. Especially if albumin-adjusted total calcium levels are >2.85 mmol/L (>11.42 mg/dL).
- Surgery should be planned in the second trimester.

#### Q3 (PHPT) – What is the preferred surgical approach for PTX during pregnancy?

- In sporadic PHPT, minimally invasive PTX in combination with intraoperative PTH monitoring.
- Bilateral neck exploration may be needed in hereditary forms.

#### Q4 (PHPT) – Which parathyroid imaging modalities should be used in pregnant women with PHPT and indication for surgery?

- Two different imaging methods should be aimed for, preferably ultrasonography in combination with either 99mTc-MIBI scan or 4D-dynamic contrast-enhanced MRI, but 18F-Fluorcholine PET/CT or methionine PET/CT may also be considered in selected cases.

#### Q5 (PHPT) – What are important considerations for the postpartum and lactation period for the mothers with PHPT and their newborns?

- Newborns are at an increased risk of hypocalcemia and hypocalcemic seizures.
- Ionized calcium levels should be measured in the newborns at least every 2 days starting on day 2 until 1–2 weeks of life.
- Consider active vitamin D treatment in case of hypocalcemia in the newborns.
- Mothers of newborn babies are at increased risk of maternal hypercalcemic crisis.
- During lactation, maternal calcium levels and eGFR should be measured every 4–8 weeks.
- PTX should be performed a few weeks after delivery.

#### Q6 (HypoPT) – What are the treatment targets and surveillance recommendations during pregnancy in women with chronic HypoPT?

- Aim to keep calcium levels (ionized or albumin-adjusted) in the lower end of the normal reference interval.
- It is difficult to predict precise doses of calcium and active vitamin D needed.
- Monitor calcium, phosphorus, eGFR, and magnesium every 3–4 week, and weekly within 1 month before giving birth.
- If changes in the doses of calcium and/or active vitamin D are needed, perform monitoring even more frequently.
- Maintain circulating 25(OH)D levels in the reference range.

#### Q7 (HypoPT) – What are the required doses of calcium and active vitamin D during pregnancy in women with chronic HypoPT?

- There is a wide variation in the required doses of calcium and active vitamin D during pregnancy.
- Dose requirements may change during the course of pregnancy.

#### Q8 (HypoPT) – Is treatment with PTH analogs and hydrochlorothiazide safe during pregnancy?

- No.

#### Q9 (HypoPT) – How to manage chronic HypoPT during lactation?

- Monitoring maternal calcium levels weekly within the first month after delivery and then every 4 weeks.

#### Q10 (HypoPT) – What are the surveillance and treatment recommendations for newborns of mothers with HypoPT?

- Newborns should be monitored clinically with ionized calcium concentrations measured every second day for the first week of life.
- Regular native vitamin D supplementation is recommended.

## Perspectives

Based on expert opinion and an interdisciplinary approach, this review attempts to address open questions, diagnostic pitfalls, and subjects of controversy, focusing on the entities of PHPT, chronic HypoPT in adults, and parathyroid disorders during pregnancy and lactation, with emphasis on unmet needs of importance for daily modern patient care.

Special attention is required to differentiate PHPT from FHH, the particularities around its milder form normocalcemic PHPT, and the management of PHPT in relation to syndromes or recurrence. The natural history of fractures, cardiovascular risk, and QoL in PHPT is also controversial and need to be covered in prospective studies. With new accumulating knowledge, evidence-based clinical guidelines should be considered.

HypoPT, a designated orphan disease, is associated with a high risk of symptoms and complications. Most cases are secondary to neck surgery, but other causes including autoimmune, genetic, or idiopathic forms also exist. Since the main cause for chronic HypoPT in adults is secondary to neck surgery, there is a need for a better understanding of predisposing factors, putative avoidance strategies, as well as optimal medical management. The recent FDA and EMA approval of rhPTH(1–84) constitutes a milestone in the management of HypoPT. It provides the natural hormone that these patients are lacking and allows for significant reductions in calcium and active vitamin D treatment. Recent evidence also suggests that QoL might be improved by substitution therapy. Questions remain regarding ideal dosing and administration regimens for rhPTH(1–84) and its long-term effects on calcium homeostasis and safety, while longer-acting PTH formulations are emerging.

Regarding pregnancy and lactation, the majority of data on diagnosis and treatment derive from isolated case reports and case series leading to low level of evidence and confidence in recommendations, especially with regard to management. An interdisciplinary approach to care combining the expertise of endocrinologists, gynecologists, pediatricians, endocrine surgeons, and nursing staff is advised. Although challenging, there is a need for prospective studies in parathyroid diseases during pregnancy and lactation, in order to improve patient care.

Acknowledging the need for further education and knowledge dissemination on parathyroid diseases within the circles of young endocrinologists in training, general practitioners, surgeons, pediatricians, and gynecologists, the ESE Educational Program of Parathyroid Disorders, PARAT 2021 aspires to deliver further educational materials in addition to the current review. These will comprise a series of webinars reflecting the conclusions of the consensus statement, slide sets, short videos, quick view guides, and a digital directory of linked resources that will be available from ese-hormones.org. Further, it is our aim to develop education material for patients, for example, patient leaflets.

## Declaration of interest

The authors declare that there is no conflict of interest that could be perceived as prejudicing the impartiality of the research reported.

## Funding

This work did not receive any specific grant from any funding agency in the public, commercial, or not-for-profit sector, besides that the PARAT program of activities 2020–2021 was supported by the European Society of Endocrinology (ESE) applying for and receiving an independent educational grant from Takeda Pharmaceutical Company and Ascendis Pharma AS. Takeda and Ascendis have not had any opportunity to influence the agenda, planned activity schedule, choice of faculty, participants, delivery formats, distribution profile of outcomes, scope of objectives, or any other kind of engagement with the Steering Group or ESE Focus Area leads.
